# Infection Patterns and Fitness Effects of *Rickettsia* and *Sodalis* Symbionts in the Green Lacewing *Chrysoperla carnea*

**DOI:** 10.3390/insects11120867

**Published:** 2020-12-07

**Authors:** Rebekka Sontowski, Michael Gerth, Sandy Richter, Axel Gruppe, Martin Schlegel, Nicole M. van Dam, Christoph Bleidorn

**Affiliations:** 1 German Centre for Integrative Biodiversity Research (iDiv) Halle-Jena-Leipzig, 04103 Leipzig, Germany; rebekka.sontowski@idiv.de (R.S.); schlegel@uni-leipzig.de (M.S.); nicole.vandam@idiv.de (N.M.v.D.); 2 Institute of Biodiversity, Friedrich-Schiller-University, 07743 Jena, Germany; 3 Department of Biological and Medical Sciences, Oxford Brookes University, Oxford OX3 0BP, UK; mgerth@brookes.ac.uk; 4 Department of Basic and Clinical Neuroscience, King’s College London, 5 Cutcombe Road, London SE5 9RT, UK; sr.sandyrichter@gmail.com; 5 Institute of Biology, Molecular Evolution and Systematics of Animals, University of Leipzig, 04109 Leipzig, Germany; 6 Chair of Zoology—Entomology Group, Technical University of Munich, 85354 Freising, Germany; gruppe@wzw.tum.de; 7 Animal Evolution and Biodiversity, Georg-Augustus-University, 37073 Göttingen, Germany

**Keywords:** biological pest control, co-infection, endosymbiont, Neuroptera, Rickettsiales, symbiosis

## Abstract

**Simple Summary:**

Bacteria have occupied a wide range of habitats including insect hosts. There they can strongly affect host physiology and ecology in a positive or negative way. Bacteria living exclusively inside other organisms are called endosymbionts. They often establish a long-term and stable association with their host. Although more and more studies focus on endosymbiont–insect interactions, the group of Neuroptera is largely neglected in such studies. We were interested in the common green lacewing (*Chrysoperla carnea*), a representative of Neuroptera, which is mainly known for its use in biological pest control. We asked ourselves which endosymbionts are present in these lacewings. By screening natural and laboratory populations, we found that the endosymbiont *Rickettsia* is present in all populations but the symbiont *Sodalis* only occurred in laboratory populations. We were curious whether both endosymbionts affect reproduction success. Through establishing and studying green lacewing lines carrying different endosymbionts, we found that *Rickettsia* had no effect on the insect reproduction, while *Sodalis* reduced the number of eggs laid by lacewings, alone and in co-infections with *Rickettsia*. The economic and ecological importance of green lacewings in biological pest control warrants a more profound understanding of its biology, which might be strongly influenced by symbionts.

**Abstract:**

Endosymbionts are widely distributed in insects and can strongly affect their host ecology. The common green lacewing (*Chrysoperla carnea*) is a neuropteran insect which is widely used in biological pest control. However, their endosymbionts and their interactions with their hosts have not been very well studied. Therefore, we screened for endosymbionts in natural and laboratory populations of *Ch. carnea* using diagnostic PCR amplicons. We found the endosymbiont *Rickettsia* to be very common in all screened natural and laboratory populations, while a hitherto uncharacterized *Sodalis* strain was found only in laboratory populations. By establishing lacewing lines with no, single or co-infections of *Sodalis* and *Rickettsia*, we found a high vertical transmission rate for both endosymbionts (>89%). However, we were only able to estimate these numbers for co-infected lacewings. *Sodalis* negatively affected the reproductive success in single and co-infected *Ch. carnea*, while *Rickettsia* showed no effect. We hypothesize that the fitness costs accrued by *Sodalis* infections might be more tolerable in the laboratory than in natural populations, as the latter are also prone to fluctuating environmental conditions and natural enemies. The economic and ecological importance of lacewings in biological pest control warrants a more profound understanding of its biology, which might be influenced by symbionts.

## 1. Introduction

With about 6000 species Neuroptera represents a rather small group of insects [1]. One well-known representative of the Neuroptera is the common green lacewing *Chrysoperla carnea*. Originally assumed to represent a single species [2], *Ch. carnea* was shown to be a complex of several cryptic species [3]. The adults feed on honeydew and pollen while the larvae are predators of a broad range of insects, e.g., aphids, mealybugs and other soft-bodied species [4,5]. Fittingly, lacewing larvae are used as efficient biological pest control agents in the field, greenhouses and orchards [5,6]. Biological pest control has received much attention through increasing insecticide resistance of several pests and legislation that aims to reduce the usage of synthetic chemical pesticides [7]. Because of their usefulness in pest control and their high resistance against many widely used pesticides, lacewings are mass-reared and marketed commercially [5,8].

Endosymbionts are widely distributed in insects and often establish a long-term and stable association with their hosts. These associations may affect host fitness through various interactions, e.g., by providing essential amino acids or vitamins, reproductive manipulation, or color modifications [9,10,11,12]. Endosymbionts are not always fixed to a specific host, several endosymbionts shift host across species [13]. One of the most common endosymbionts in insects is *Rickettsia* spp. (α-Proteobacteria) with an estimated distribution in one-quarter of all terrestrial arthropod species [14]. The endosymbiont *Rickettsia* spp. was first described in randomly sampled arthropod host screening which also included Neuroptera [15,16]. *Rickettsia* spp. can also infect vertebrates, leeches, freshwater polyps, unicellular eukaryotes and plants [17,18,19,20,21]. *Rickettsia* strains that infect vertebrates, such as human pathogens, are all transmitted by arthropods and the majority of *Rickettsia* lineages seem to be restricted to arthropods [15,17]. Some of them alter host reproduction by causing male-killing (e.g., in some ladybird beetles (Coccinellidae) or jewel beetles (Buprestidae)), or parthenogenesis (e.g., in eulophid wasps) [22,23,24,25]. *Rickettisa* spp. are abundant in natural and laboratory insect populations, establish themselves rapidly, and remain stable at high frequencies in populations [26,27].

The role of endosymbionts in Neuroptera has so far been largely neglected. Two recent studies have described the endosymbiont *Spiroplasma* in the green lacewing *Mallada desjardinsi* with a male-killing phenotype [28,29]. Recently, a Neuroptera-specific *Rickettsia* screening showed that approximately 40% of the tested Neuroptera species were infected with *Rickettsia*, including *Ch. carnea* [30]. In the following sections, we will refer to the species *Ch. carnea* s.str. as *Ch. carnea*. This species was infected by strains of the *R. bellii* clade, commonly found in arthropods [15,30]. While screening *Ch. carnea* for endosymbionts, we also found infections with *Sodalis* spp., a facultative symbiont belonging to the γ-proteobacteria [31]. *Sodalis* was first identified in tsetse flies and later detected in different insect groups such as weevils, stinkbugs, louse flies and lice [32,33,34,35,36]. The prevalence of *Sodalis* infections can vary greatly in arthropods [37], as can the reported host–*Sodalis* interactions [38]. This symbiont is able to facilitate trypanosome infections in tsetse flies (Glossinidae), participate in the cuticle synthesis of weevils and modify host phenotypes [35,39]. Interestingly, a low density of *Sodalis* in weevils produces a host killing phenotype, whereas a high prevalence leads to a persistent and beneficial infection in the hosts [40].

Although green lacewings are intensively used in biocontrol applications, it is still unclear how endosymbionts are distributed on species and population level and their role in these hosts. In the present study, we screened several natural populations of *Ch. carnea* for *Rickettsia* and *Sodalis* infections. We also tested the similarity of infection patterns in natural and laboratory populations. To characterize the *Sodalis* symbiont in *Ch. carnea,* we assembled a draft genome from Illumina short reads for subsequent phylogenomic analysis. To study host–endosymbiont interactions, we generated *Ch. carnea* lines that carried *Rickettsia,* or *Sodalis,* or both, as well as endosymbiont-free lines. Based on these lines, we examined the potential impact of endosymbionts on host reproduction and the rate of vertical transmission for both symbionts in double infected lacewings. We demonstrate that the *Ch. carnea*/*Rickettsia*/*Sodalis* system represents a promising model for the evolution of insect–endosymbiont interactions in general.

## 2. Materials and Methods

### 2.1. Endosymbiont Screening in Natural and Laboratory Ch. carnea Populations

To compare the infection rate of endosymbionts under natural and laboratory conditions, we obtained *Ch. carnea* individuals from three different commercial suppliers (*n* = 64 in total) and sampled eight natural populations (*n* = 84 in total). The supplying companies were Sautter and Stepper GmbH (Ammerbuch-Altingen, Germany, 26 larvae), Biobest (Westerlo, Belgium, 18 larvae) and Katz Biotech AG (Baruth/Mark, Germany, 20 larvae). Samples from natural populations were collected in various locations in Germany and Austria between 2010 and 2015 (Table S1) and transported and stored in 70% ethanol. Species were identified based on morphology. Genomic DNA was extracted from whole insects using the NucleoSpin Tissue Kit (Marchery-Nagel, Düren, Germany) by following the manufacturer’s instructions. The quality and quantity of extracted DNA were investigated using gel electrophoresis (1% agarose gel) and a Nanophotometer P330 (Implen, Munich, Germany). To identify a *Rickettsia* and/or *Sodalis* infection, a PCR screening with species-specific *16S* rRNA gene primer (Table S2) was performed. Fragments of the *16S* rRNA gene were amplified using 2 µL dNTPs, 2.5 µL DreamTaq Green Buffer, 1 µL of the corresponding forward and reverse 10 µM primer, 12.5 µL HPLC water, 0.1 µL DreamTaq™ Green DNA polymerase (Thermo Fisher Scientific, Waltham, MA, USA) and 1 µL of template DNA. A fragment of *Rickettsia 16S* rRNA gene was amplified at 95 °C for 2 min, followed by 30 cycles of 92 °C for 30 s, 58 °C for 30 s and 72 °C for 30 s, and a final extension at 72 °C for 5 min [41]. A fragment of *Sodalis 16S* rRNA gene was amplified at 95 °C for 10 min, followed by 35 cycles of 94 °C for 30 s, 55 °C for 60 s and 72 °C for 90 s (according to [42] with minor adaptations). PCR amplicons were counted as positive evidence for *Rickettsia* or *Sodalis* infection. To exclude false negatives (e.g., due to low titer of the endosymbiont), the PCR product was diluted to 1:10, 1:100, and 1:1000. These dilutions were subjected to PCR again. When no bands were visible for any of the PCRs, the sample was counted as not infected. We included in each run per primer pair a negative control containing all PCR ingredients except for the DNA, and a positive control containing a DNA sample that was previously tested positive.

### 2.2. Molecular Characterization of Endosymbionts

We used a whole-genome shotgun approach to generate a metagenome assembly containing host and endosymbiont contigs for data mining. For this purpose, a double-indexed Illumina library was constructed from the DNA of a co-infected 2nd instar green lacewing larva as detailed in Meyer and Kircher [43] and Kircher et al. [44]. The insect sample was obtained from the company Sautter and Stepper GmbH (Ammerbuch-Altingen, Germany) and DNA extracted using the DNeasy^®^ Blood and Tissue Kit (Qiagen, Venlo, The Netherlands) following the supplier’s instruction. The quality of the extracted DNA was tested with a Nanophotometer and gel electrophoresis (1% agarose). The library was then sequenced as 140-bp paired-end run on an Illumina HighSeq 2500 (Illumina, San Diego, CA, USA) at the Max Planck Institute for Evolutionary Anthropology (Leipzig, Germany). Base-calling was performed with freeIbis [45], adapters were trimmed and reads with more than five bases below a quality threshold of 15 were discarded. A meta-assembly was created using IDBA-UD [46], with k-mers 21–81 in steps of ten. Markers for qPCR and molecular characterization of endosymbionts were recovered using BLAST-searches against the assembled contigs. Raw reads have been submitted to NCBI Genbank under the accessions PRJNA506348. The assembly is available under https://doi.org/10.5281/zenodo.2163010. *Rickettsia* endosymbionts of green lacewings were already phylogenetically characterized in Gerth et al. [30]. For the molecular characterization of the *Sodalis* strain, assembled contigs were blasted with BLASTN against NCBI GenBank. All recovered *Sodalis* contigs were used as reference for subsequent mapping using NextGenMap 0.4.12 [47] to retrieve all putative *Sodalis* reads. The coverage of all *Sodalis* contigs was evaluated with qualimap 2.2.1 [48] and retrieved reads were newly assembled with SPAdes 3.1.1 [49], an assembler optimized for bacterial and archaeal genomes, to generate a *Sodalis* draft genome. For the phylogenetic placement of the lacewing *Sodalis* strain, we recovered the *16S* rRNA gene using BLAST search and compiled a *Sodalis* reference dataset based on a recent study by Sochova et al. [38]. A list of sequences included in the analysis can be found in Table S3.

The alignment was conducted using MAFFT [50]. We used GUIDANCE2 [51] for alignment masking, where we chose to generate alternative guide trees from 100 bootstrap replicates. After the removal of unreliable columns, we additionally removed all columns containing more than 20% gaps. For phylogenetic analyses, we conducted model testing and a Maximum Likelihood analysis with 1000 bootstrap replicates using IQ-TREE version 1.4.2 [52]. The selected model was the TIM3 + I + G model, with the three rate categories.

### 2.3. Endosymbiont–Host Interaction

#### 2.3.1. Insect Rearing

Total developmental time was approximately 70 days from eggs to adults in *Ch. carnea* under our laboratory conditions. Insect cultivation and all experiments were carried out under controlled environmental conditions in a climate cabinet (Percival Scientific, Perry, IA, USA) with a constant temperature of 22 °C ± 2 °C, a 16 h light and 8 h dark cycle, and relative humidity of 65 ± 5%. We started a *Ch. carnea* culture with 85 second instar larvae obtained from the company Sautter and Stepper GmbH (Ammerbuch-Altingen, Germany). All larvae were reared individually in small round plastic containers (3 cm diameter) and fed with dead moth eggs (*Sitotroga* sp., Katz Biotech, Baruth/Mark, Germany) twice a week. Water droplets were added to the container wall three times a week. To exclude contaminations from the diet, *Sitotroga* eggs were PCR screened for *Rickettsia* and *Sodalis* infections, which were not detectable in the diet. At the 3rd instar, a small piece of corrugated cardboard was added to each container to facilitate pupation. After eclosion, adult lacewings were fed with a mixture of honey, water, yeast extract, and sucrose (1:1:1:1) every other day. Adults aged 7 days were then mated by putting 2–4 females and 2–4 males in one cage (38 × 38 × 60cm). After 5 days, females were separated into a plastic container (~38 cm^3^), covered with a fine cotton mesh to encourage oviposition and fed every other day with the food mixture as previously described. The mesh containing eggs was changed every 5 days and stored in a small petri dish (6 × 1.5 cm). These dishes were checked daily and hatched larvae were collected and reared separately to reduce the rates of cannibalism. After eggs were collected, all mothers were screened via PCR for the presence of *Sodalis* and *Rickettsia* as previously described. This approach allowed us to establish lines of lacewings that were either (1) symbiont free, (2) infected with *Rickettsia* only, (3) infected with *Sodalis* only or (4) infected with both *Rickettsia* and *Sodalis*. All further experiments were performed after amplifying the lines for two generations.

#### 2.3.2. Vertical Transmission Rate of Endosymbionts in *Ch. carnea*

Vertical transmission rates of both endosymbionts were studied in 14 *Ch. carnea* females with a combined infection of *Rickettsia* and *Sodalis*. For this purpose, females were mated with males carrying *Rickettsia* and *Sodalis* and reared as previously described. After 16 days the females were removed and PCR screened for *Rickettsia* and *Sodalis* symbionts as described above. Offspring were collected every day and allowed to develop for 28 days (until 3rd instar), before being collected and PCR screened for the symbionts. This was done for practical reasons, as it is difficult to reliably extract DNA from single individuals in earlier stages. The rate of vertical transmission was determined by calculating the number of infected offspring divided by the number of total offspring tested per female. Based on the low number of single *Rickettsia* and *Sodalis* infected individuals in the ordered larvae, we were not able to get a representative number of replicates for these three lines to study vertical transmission in single infected green lacewings.

#### 2.3.3. Effect of Endosymbionts on Host Reproductive Success

The endosymbiont effect on host reproductive success was studied in 30 female lacewings, of which 7 were infected with *Rickettsia* only, 8 with *Sodalis* only, 10 with *Rickettsia* and *Sodalis* and 5 were without any of these symbionts. *Rickettsia*, *Sodalis*, and co-infected females were mated with males of the same infection status. In combined endosymbiont pairs, 4 males were singly infected with *Sodalis*. Females without symbionts were mated with *Rickettsia* or *Rickettsia* and *Sodalis* infected males to control for endosymbiont-mediated sex bias. Non-conformities in the infection stages of the males were based on endosymbiont screening after mating. After a mating period of five days, adults were separated and females were placed individually in round plastic containers. We counted the number of eggs per female every five days for 45 days in total and transferred all eggs from one female into a small petri dish. After these 45 days, the mothers were killed and screened for symbionts by PCR as described above. Starting from the day the first eggs were collected, we visually inspected the Petri dishes for hatched larvae. The larvae were counted visually every day until all eggs were empty or dried out. All larvae were then kept separately in small cups and fed with dead *Sitotroga* eggs until they pupated. The number of pupae and emerged adults were counted every day (Table S4). Finally, using a general linear model with a quasi-Poisson distribution with the standard commands in R [53], we compared the reproductive success for the category “number of eggs” among the four different endosymbiont lines. We used the same statistical model for the categories, “larvae”, “pupae” and “emerged adults”.

#### 2.3.4. Endosymbiont Titer in Single and Co-Infected *Ch. carnea*

To determine symbiont titers in single and co-infected green lacewings, we used a qPCR approach. To this end, we collected 26 adult females (9 with *Rickettsia* only, 9 with *Sodalis* only, 8 with *Rickettsia* and *Sodalis*). All of them were unmated and of similar age (~14 days). In addition, we tested *Rickettsia* and *Sodalis* titer in 10 co-infected larvae. Due to the low percentage of single infected individuals, we were unable to collect a representative number of only *Rickettsia* or *Sodalis* infected larvae. Genomic DNA was extracted using the NucleoSpin Tissue Kit protocol as previously described. Quantification of *Rickettsia* and *Sodalis* was examined by amplification of a 222 bp fragment of *gltA* for *Rickettsia* and a 182 bp fragment of *groEL* for *Sodalis*, respectively. To design *Rickettsia* and *Sodalis* specific primers, we used a published *R. bellii gltA* sequence for *Rickettsia*, which was available in NCBI (accession number DQ146481.1) and for *Sodalis* a *groEL* sequence of the closely related *S. praecaptivus* (accession number JX444566.1). Both sequences were blasted with BLASTN against our whole-genome shotgun assembly (see above) and the corresponding contigs were aligned with MAFFT version 7 [50]. Primers for these fragments were designed using Primer3 v. 0.4.0 (Table S2, [54]). Primer efficiency was tested using a 1:10 dilution series of DNA from pooled co-infected *Ch. carnea* adults. The calculated efficiency was similar for all qPCR primers (105–106%). To normalize to host size, we amplified a fragment of the host insect gene *actin*, using primers from Liu et al. [55]. *Actin* was tested for an almost constant Ct value between treatments by screening 30 *Rickettsia*, *Sodalis* or *Rickettsia* and *Sodalis* infected *Ch. carnea* (STD ± 0.5 cycles).

All qPCR reactions were performed in 10 µL volumes on a PikoReal Real-time PCR System (Thermo Fisher Scientific, Waltham, MA, USA) using 5 µL Maxima SYBR^®^ Green qPCR Master Mix (2X, Thermo Fisher Scientific, Waltham, MA, USA), 2.5 µmol of forward and reverse primer and 500ng DNA. The qPCR program was set as follows: initial incubation at 95 °C for 1 min, followed by 40 cycles at 95 °C for 15 s, 55 °C for 15 s, and 72 °C for 45 s, followed by a 0.2 °C increment melt curve from 60 to 95 °C. After PCR amplification, a melt curve analysis was conducted using the PikoReal Software (Thermo Scientific, Waltham, MA, USA) to confirm that there was one amplified product. Individual samples were run in duplicates. A no-template control and negative control for both endosymbiont primer sets were run on each plate. The negative control contained DNA of *Ch. carnea* only infected with the endosymbiont where the primer was not designed for, for example, we used the DNA of an only *Sodalis* infected *Ch. carnea* individual for the primer set of *gltA* (*Rickettsia*). The negative controls and the no-template controls did not show amplification for any of the genes tested.

To compare endosymbiont density in single and co-infected adults and co-infected larvae, we used gene copies as a proxy for symbiont number in relation to copies of a host gene as a normalization of host size. This was done by calculating the 2^ΔCt^ values using the Ct value of the corresponding endosymbiont and host gene. We refer to this value in our manuscript titer. Differences between groups were determined using a one-way ANOVA with Tukey post-hoc test in R [53]. For statistical analysis, the calculated values of all genes were normalized by using a log transformation (Table S5).

## 3. Results

### 3.1. The Screening of Rickettsia and Sodalis Symbionts on Population Levels in Natural and Laboratory Ch. carnea

**Figure 1:**
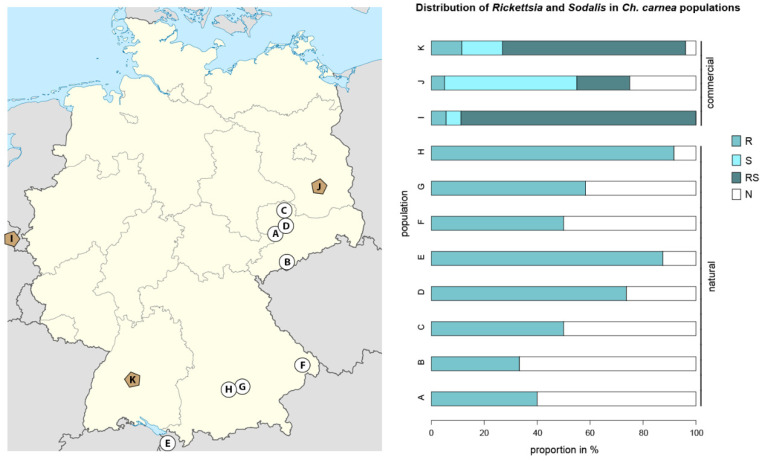
Distribution of *Rickettsia* and *Sodalis* symbionts in *Chrysoperla carnea* s. str. in natural populations (Saxony and Bavaria, Germany and Austria; map: white circle) and commercially reared populations (map: dark rhombus indicates the location of the company headquarters, not the origin of populations). R: *Rickettsia* infected, S: *Sodalis* infected, RS: *Rickettsia* and *Sodalis* infected, N: uninfected. Letters in the map highlight collection/rearing sites. A: Trages, B: Neudorf, C: Dahlen, D: Püchau, E: Rankweil, F: Schönberg, G: Wippenhausen, H: Kranzberg, I: Biobest, J: Katz Biotech, K: Sautter and Stepper.

To compare distribution patterns of endosymbionts in *Ch. carnea* in natural and laboratory populations, we screened 148 individuals representing eight natural and three commercially propagated populations. *Rickettsia* infections were found in all-natural populations; in total, 64% of the individuals were infected (33–92% infected individuals/population, Figure 1). In commercial laboratory populations, *Rickettsia* was found in 67% of all screened individuals (25–94% infected individuals/population), 12% of these as single infections and 88% as co-infections with *Sodalis*. The endosymbiont *Sodalis* was only detected in laboratory *Ch. carnea* where 83% of screened individuals were infected (70–94% infected individuals/population), 28% of these as single infections and 72% as co-infections with *Rickettsia* (Figure 1).

### 3.2. Molecular Phylogenetic Characterization of Endosymbionts

Previous phylogenetic analysis demonstrated that the *Rickettsia* strain that infected *Ch. carnea* belonged to the *Rickettsia bellii* clade [30]. For the *Sodalis* strain in *Ch. carnea*, 4,289,304 reads could be used to assemble a draft genome, which was represented by 558 contigs with an N50 of 20,104 b and a coverage of ~67x. Based on this draft, the genome of the *Sodalis* endosymbiont was around 4.3 Mbp in size.

**Figure 2:**
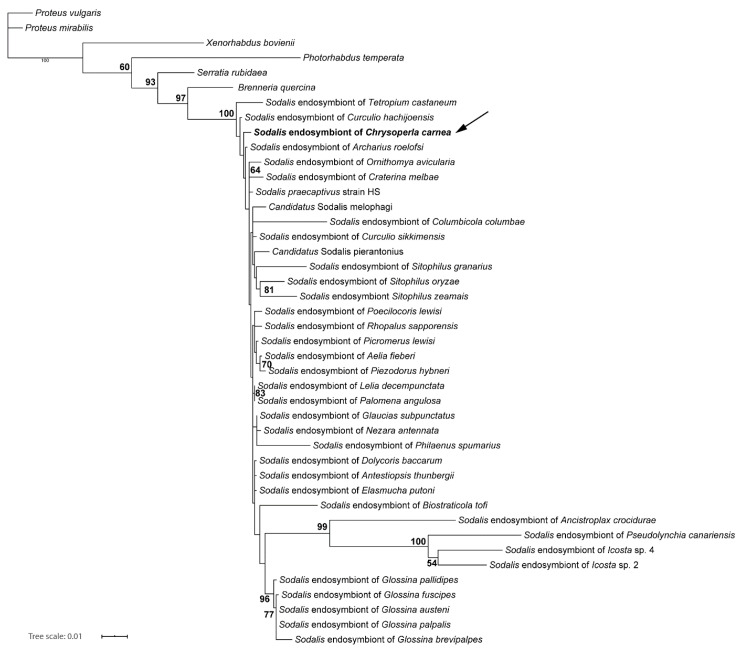
Phylogenetic analysis of the 16S rRNA gene dataset of *Sodalis* strains using Maximum Likelihood under the TIM3 + I + G model as implemented in IQ-TREE. Bootstrap support from 1000 pseudoreplicates is given at the nodes. Only support values higher than 50% are displayed.

For the phylogenetic placement using the 16S rRNA gene, our analyses found the green lacewing *Sodalis* strain nested within a strongly supported monophyletic group of *Sodalis* endosymbionts (Figure 2). This clade contains mostly symbiotic bacteria isolated from insect hosts, but also *Sodalis praecaptivus* isolated from human wound tissue. The *Ch. carnea* infecting strain was found in a rather basally branching position, branching between some lineages isolated from beetle taxa, but not forming a clade with them. Altogether, the ingroup topology of *Sodalis* was not well supported and partly not resolved.

### 3.3. Endosymbiont Host Interaction

The rate of vertical transmission estimated from the number of infected offspring divided by the number of total offspring was overall very high for both endosymbionts (>89%). However, *Sodalis* was vertically transmitted at a slightly higher rate (97%) than *Rickettsia* (89%) in co-infected lacewings (Table 1).

**Figure 3:**
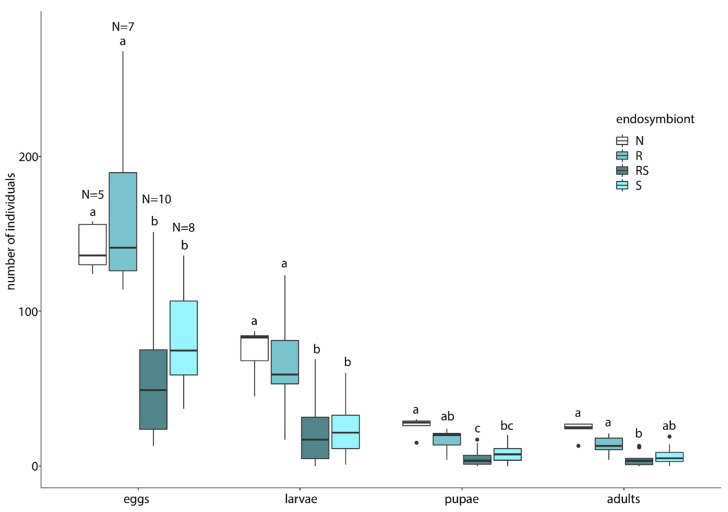
Number of eggs, larvae, pupae and adults of *Chrysoperla carnea* s. str. lines differing in levels of endosymbiont infections. N: non-infected, R: only *Rickettsia*, S: only *Sodalis* or RS: co-infected with *Rickettsia* and *Sodalis*. Values are presented as boxplot and the box is defined by the 25th and 75th percentiles (lower and upper quartile). The thick horizontal line is the median and the vertical lines (whiskers) have maximum a distance of 1.5 times the percentiles length. Dots outside the line can be considered as outliers. Different letters indicate *p*-values < 0.05 based on general linear model, *n* = number of females.

Reproductive success differed considerably between groups. We found a reduced reproductive output in single *Sodalis* infected *Ch. carnea* in comparison to uninfected lacewings in all life stages (Figure 3, Table 2). Single *Rickettsia* infected *Ch. carnea* performed similarly as uninfected lacewings. Lacewings with co-infections of *Rickettsia* and *Sodalis* showed a reduced reproductive output similar to single *Sodalis* infections. In general, the number of hatched larvae was rather low, which might indicate that our rearing conditions were sub-optimal (Figure 3, Table S4).

**Figure 4:**
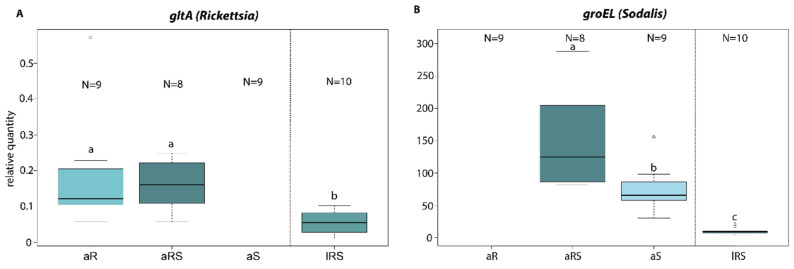
Relative quantity of *Rickettsia* and *Sodalis* endosymbionts in *Chrysoperla carnea* s. str. using *Rickettsia* specific (*gltA*) (**A**) or *Sodalis* specific (*groEL*) gene fragments (**B**). *Ch. carnea* were infected with only *Rickettsia* or *Sodalis* or co-infected. a: adult; l: larvae; S: *Sodalis* infection only; R: *Rickettsia* infection only; RS: co-infection with *Rickettsia* and *Sodalis*; Values are presented as boxplot and the box is defined by the 25th and 75th percentiles (lower and upper quartile). The thick horizontal line is the median and the vertical lines (whiskers) have maximum a distance of 1.5 times the percentiles length. Dots outside the line can be considered as outliers. Different letters indicate significant differences between groups using one-way ANOVA, *p*-value ˂0.05.

Finally, by using qPCR we found that the *Rickettsia* titers were similar between single and co-infected adult individuals of *Ch. carnea* (Figure 4). Larvae–adult comparison of *Rickettsia* titer in co-infections significantly differed, with the larvae having a lower titer (Figure 4, Table 3). *Sodalis* titers differed significantly in single and co-infected adults. Co-infected adults with *Rickettsia* had a distinctly higher *Sodalis* titer than single infected ones. The *Sodalis* titer was also reduced in co-infected larvae compared to co-infected adults.

## 4. Discussion

### 4.1. Endosymbiont Screening on Population Levels in Natural and Laboratory Ch. carnea

Our analysis showed that both *Rickettsia* and *Sodalis* were common endosymbionts in *Ch. carnea* populations. This is the first record of *Sodalis* sp. found in Neuroptera. However, the most common endosymbiont was *Rickettsia*, which occurred in all tested natural and laboratory populations. Our screening of *Rickettsia* infections revealed infection rates ranging from 25% to 94% in both population types (laboratory and natural). The highly variable infection rates between populations are typical for *Rickettsia* infections in insects. For instance, wild whitefly (*Bemisia tabaci*, Hemimptera: Aleyrodidae) populations showed a very broad range in *Rickettsia* infection frequency (22% to 100%). In Buprestidae (Coleoptera) approximately half of the tested individuals were infected (46.3%) and in the mirid bug species *Nesidiocoris tenuis* (Heteroptera: Miridae) nearly all individuals were infected (93% to 100%) [23,41,56].

While screening green lacewings for endosymbionts using a metagenomic approach, we also detected *Sodalis,* a well-known γ-proteobacterial endosymbiont of tsetse flies [57]. This symbiont was also found in several other insects, such as stinkbugs, spittlebugs, bird lice, hippoboscid flies, weevils, psyllids or scale insects. [36,37,58]. Although we detected a high frequency of *Sodalis* symbionts in *Ch. carnea* in all laboratory populations, none was found in natural ones (Figure 1). Differences in the presence of *Sodalis* in commercially available specimens and naturally collected specimens were also noticed by Saeed and White in bees [59]. They observed *Sodalis* in only 3 out of 100 individual bees captured in the wild and in 10 out of 85 individuals when sampling commercially reared individuals. Variations in endosymbiont infection rates in natural and laboratory populations seem to be host species-specific. While *Wolbachia* infection rates were similar in natural and laboratory *Drosophila melanogaster* (Diptera) populations [60], this was not true for tsetse flies. The same *Wolbachia* supergroup had highly variable infection rates in natural populations, whereas 100% of the individuals were infected in laboratory populations of the tsetse fly *Glossina morsitans morsitans* [61].

The complete absence of *Sodalis* in natural *Ch. carnea* populations is potentially caused by differences in selection pressures between laboratory and natural populations. In natural populations, lacewings are subject to fluctuating environmental conditions, natural enemies and competition for nutrition with other arthropods. Conceivably, these additional sources of stress are more relaxed or missing under laboratory conditions, which may be favorable for *Sodalis*. In line with this, our data suggest that *Sodalis* infections cause fitness costs in *Ch. carnea.* Based on the fitness effects, *Sodalis* may be eliminated faster in natural than in laboratory populations, where inbreeding and stable conditions may enhance the transmission rate of endosymbionts. For instance, temperature varies greatly under natural conditions, whereas laboratory populations are generally reared at a constant and relatively high temperature to optimize breeding success. A correlation of temperature with the rate of transmission has been reported for several bacteria [62,63]. A higher *Sodalis* infection frequency was detected in weevils living at localities with higher temperatures [64]. It is also conceivable that *Sodalis* reinfections from the environment occur more frequently in laboratory populations. A further reason for the absence of *Sodalis* in natural populations might be our sampling design. Due to the low number of sampled individuals per population and our focus on two regions (Saxony and Bavaria/Austria), we might have missed *Sodalis* in case of a low prevalence in the population. A low prevalence of *Sodalis* in natural populations of insect hosts has been described from stinkbug populations (7.5%) [37]. If individuals from such *Ch. carnea* populations were sampled for commercial breeding and based on the efficient vertical transmission of this symbiont, we would find a high *Sodalis* frequency in commercial populations as our data showed. However, given the current state of knowledge, it can only be speculated why *Sodalis* is only present in laboratory populations of the tested green lacewings.

### 4.2. Molecular Phylogenetic Characterization of Endosymbionts

Both *Rickettsia* and *Sodalis* symbionts occur in many diverse arthropod orders and often different strains infect different hosts. [15,37,38,57]. *Rickettsia* is subdivided into 13 lineages. In general, they do not co-speciate with their hosts, however, a host shift takes place more frequently between related arthropods [15]. A study that investigated *Rickettsia* strains in the insect order Neuroptera, to which our host insect belongs, found also a more host-specific distribution of diverse *Rickettsia* lineages in this insect order [30]. Our finding supported this prediction. We found the same strain (*R. bellii* group) in the here investigated green lacewings as Gerth et al. [30] already reported for different populations of *Ch. carnea*.

To classify the *Sodalis* symbiont of *Ch. carnea* also in terms of their evolution, this endosymbiont was characterized genetically. Our phylogenetic analysis showed that this strain is closely related to published *Sodalis* strains. Several other close relatives of this species have already been identified in different insect hosts, such as Coleopetera or Heteroptera [65,66]. Whereas almost all other known *Sodalis* strains have been described as primary or secondary endosymbionts of insects, we also recovered *Sodalis praecaptivus* nested between these strains. *S. praecaptivus* was isolated from a human wound as a result of an accident with a tree branch [67]. The *S. praecaptivus* strain is regarded as a free-living member of *Sodalis*. Its genome is 5.17 Mbp, the largest of all sequenced *Sodalis* strains so far [68]. With 4.3 Mbp, the draft genome of the *Sodalis* strain in green lacewings is comparable in size to those of *Candidatus* S. pierantonius (4.5 Mbp), a secondary endosymbiont of the rice weevil [69], and *S. glossinidius* (4.2 Mbp), a secondary endosymbiont of the tsetse fly [70]. The *Sodalis*-like primary endosymbiont of the spittlebug *Philaenus spumarius* has 1.39 Mbp, the smallest genome of all known strains [58,71]. It has been hypothesized that *Sodalis* strains adapted independently to an endosymbiotic lifestyle with different insect hosts, resulting in a reduction of genome size and complexity [69,72]. The genome size of the different strains seems to correlate with the level of dependency to their hosts. However, a more contiguous assembly of the green lacewing *Sodalis* strain is necessary for a detailed analysis of the state of its “genome degeneration”.

### 4.3. Endosymbiont Host Interaction

Based on the high infection rate in several *Ch. carnea* populations, the vertical transmission rate of both endosymbionts was studied in co-infected green lacewings. When sharing the same host, endosymbionts have to compete for nutrients and space either by sharing resources or evolving different niches, e.g., inhabit special cells or organs [73,74]. This phenomenon was found in tsetse flies, where *Wolbachia* only infects oocytes, *Wigglesworthia* bacteriocytes and milk glands, and *Sodalis* several organs [75]. However, our data show that also in co-infections, *Rickettsia* and *Sodalis* transmission rates are very high in green lacewings (89–97%, Table 1). The *Rickettsia* transmission rate is consistent with an earlier study in whiteflies under laboratory conditions [26]. Slightly lower rates were found in studies of tsetse flies (*Glossina morsitans*) for *Sodalis,* (67–75%, [39,76]). However, it has to be considered that the tsetse flies were infected with another *Sodalis* strain than the green lacewings. Whether both *Rickettsia* and *Sodalis* share the same host cells in green lacewings or they are spatially separated remains to be explored.

Negative fitness impacts are a prevalent phenomenon associated with endosymbionts [10]. Therefore, we studied the effects of *Rickettsia* and/or *Sodalis* on their host reproductive success in *Ch. carnea.* In the present study, we found no effect of single *Rickettsia* infections on the reproductive success of its host (Figure 3). In general, *Rickettsia* spp. are able to manipulate insect fitness in both negative or positive ways. While negatively affecting body weight, fecundity and longevity in aphids [77,78], a positive effect was found on body size, number of offspring, development and survival rate in whiteflies and leeches [19,26]. In the present study *Sodalis* seems to have a detrimental effect on the number of viable offspring in *Ch. carnea.* Starting with a lower amount of laid eggs, the number of larvae, pupae and eclosed adults were also reduced compared to endosymbiont free breeding lines. This impact on fecundity and pupal emergence rate was not found in tsetse flies. In these hosts, *Sodalis* established trypanosome infections and longevity [39,79,80]. However, it has to be considered that tsetse flies were infected with the strain *S. glossinidius*. The effect of *Sodalis* on other insects is less well understood. Detrimental effects on host fitness were also reported from other endosymbionts. The endosymbiont *Regiella* spp. inhibited the formation of winged aphids in the grain aphids *Sitobion avenae*, depending on the temperature [81]. It might be that the detrimental effect of *Sodalis* in *Ch. carena* also occurs only under certain environmental conditions. The generally low larval hatching rates in our study may indicate sub-optimal rearing conditions. This possibly leads to the fact that our results do not occur under more optimal conditions. Carrying an endosymbiont is often accompanied by a trade-off for the host. A meta-study about endosymbionts in aphids demonstrated that endosymbionts are often associated with costs for the hosts, such as increased developmental time, reduced longevity and fecundity. On the other hand, hosts often benefit from resistance against parasitic wasps [82]. It is conceivable that we missed the beneficial effects of *Sodalis* infections in *Ch. carnea*. The co-occurrence of *Rickettsia* and *Sodalis* found in *Ch. carnea* was also reported in weevils and lice [64,83]. In our study, co-infections showed a detrimental effect on reproductive success, similar to single *Sodalis* infections. To test, whether endosymbiont density is constant in single and co-infected green lacewings, *Rickettsia* and *Sodalis* titers were measured in both infection lines (single and co-infected). We found an asymmetrical interaction between the co-infected symbionts. *Rickettsia* density was not affected by co-infections with *Sodalis* endosymbionts in adult green lacewings. This indicates that even both symbionts are carried by the same host, they might have occupied different niches to avoid competition about available nutrients and space. For example, in the whitefly *Bemisia tabaci Rickettsia* was found throughout the whole host body and other endosymbionts (*Hamiltonella*, *Wolbachia* and *Cardinum*) were only localized in bacteriocytes [84]. Other studies showed that *Rickettsia* can also be localized in specific host tissues [78]. Whether *Rickettsia* is evenly distributed or localized on specific tissue in *Ch. carnea* has to be studied. Interestingly, we observed a contrasting interaction between single and co-infections of *Sodalis* in *Ch. carnea*, where single infections showed a lower density than in the presence of *Rickettsia* (Figure 4) These findings suggest that *Sodalis* might benefit from the presence of *Rickettsia*, even though we found no enhanced phenotypic effect on the reproductive success in co-infected *Ch. carnea*.

During the lifetime of *Ch. carnea* the density of both endosymbionts was not constant in co-infected individuals. We found a lower *Sodalis* and *Rickettsia* density in larvae than in adults. Variations of endosymbiont density during the life cycle of insects is not uncommon. *Sodalis* infections were also not detected in all host life stages of the cereal weevils (*Sitophilus*, Coleoptera) where they probably are involved in cuticle synthesis in young adults, after which the endosymbiont was eliminated [35]. The density of other endosymbionts also varies during the lifetime of insect hosts. For instance, the endosymbiont *Spiroplasma* in *Drosophila melanogaster* was reduced in the larval stage [74]. Reasons for the low density of both endosymbionts in *Ch. carnea* larvae might be influenced by using different diet sources in different life stages (insects vs. honeydew and pollen), different environments (living exclusively on a plant vs. flying around plants), larval-specific developmental factors (e.g., moulting) or changes in the host immune system between both life stages. Therefore, further investigation is required, especially regarding the relevance of the symbionts and their function in several life stages.

## 5. Conclusions

This work is a first step in studying the distribution and fitness impact of endosymbionts in the common green lacewing *Ch. carnea*, a species frequently used in biological pest control. Based on our results, we generated new hypotheses about underlying mechanisms and functions of this specific symbiont–host interaction. The negative fitness effect of *Sodalis* found in this study may have an important impact on commercial rearing and it should be explored if treating *Ch. carnea* with antibiotics may improve the rearing success and efficiency.

## Figures and Tables

**Table 1:** Number and percentage of offspring infected with *Rickettsia* and/or *Sodalis* symbionts from 13 double infected *Chrysoperla carnea* s. str. females (in total 104 individuals were tested).

	*Rickettsia*	*Sodalis*
Number of offspring infected with only one endosymbiont	3 (2.9%)	11 (10.6%)
Number of offspring infected with both endosymbionts	90 (86.5%)	90 (86.5%)
Total number of infected offspring	93 (89.4%)	101 (97.1%)

**Table 2:** Statistical comparison of laid eggs, viable larvae, number of pupae and emerged adults of *Chrysoperla carnea* s.str. lines of different endosymbiont infections, using a general linear model with a quasi-Poisson distribution. *n*: no endosymbiont, R: only *Rickettsia*, S: only *Sodalis,* RS: *Rickettsia* and *Sodalis* infected. *p*-value * < 0.05; ** < 0.01; *** < 0.001.

		R-N	RS-N	S-N	R-RS	R-S	RS-S
**Eggs**	t *p*-value	0.678 0.504	−3.066 0.005 **	−2.063 0.049 *	−3.739 0.001 **	−2.730 0.012 *	0.90 0.382
**Larvae**	t *p*-value	−0.327 0.747	−3.212 0.004 **	−2.941 0.007 **	−2.877 0.009 **	−2.614 0.016 *	0.069 0.945
**Pupae**	t *p*-value	−1.502 0.145	−4.276 <0.001 ***	−3.340 0.003 **	−2.856 0.009 **	−1.914 0.070	0.868 0.398
**Adults**	t *p*-value	−1.864 0.074	−4.518 <0.001 ***	−3.606 0.001 **	−2.809 0.010 *	−1.863 0.080	0.883 0.390

**Table 3:** Statistical results of the relative quantity of *Rickettsia* (*gltA)* and *Sodalis* (*groEL*) in adult *Chrysoperla carnea* s. str. with *Rickettsia* only (aR), *Sodalis* only (aS) or *Rickettsia* and *Sodalis* (aRS) infections and in co-infected larvae (lRS) using a one-way ANOVA and Tukey post-hoc test. *p*-value * < 0.05, ** <0.01, *** < 0.001.

Test	Lines	*gltA*(*Rickettsia*)	*groEL* (*Sodalis*)
ANOVA		F_(2/24)_ = 9.744	F_(2/24)_ = 146.18
		*p* < 0.001 ***	*p* < 0.001 ***
Tukey post-hoc	aS-aRS	-	*p* = 0.021 *
	aR-aRS	*p* = 0.997	-
	lRS-aRS	*p* = 0.002 **	*p* < 0.001 ***

## Supplementary Materials

The following materials are available online at https://www.mdpi.com/2075-4450/11/12/867/s1, Table S1: Collection data and endosymbiont infections of natural *Chrysoperla carnea* populations, Table S2: PCR and qPCR primer, Table S3: Accession numbers of sequences used for the phylogenetic placement of the green lacewing *Sodalis* strain, Table S4: Experimental data of reproduction effect experiment, Table S5: Relative quantity of *Rickettsia* and *Sodalis* endosymbionts in *Chrysoperla carnea*.
