# Supplementary material for: Infection Patterns and Fitness Effects of Rickettsia and Sodalis Symbionts in the Green Lacewing Chrysoperla carnea
Source: Insects. 2020 Dec 7;11(12):867. doi: 10.3390/insects11120867 (PMC7762206; doi:10.3390/insects11120867)
Supplement: Supplementary file 1 [file insects-11-00867-s001.pdf]

Supplementary material

Table S1 Collection data and endosymbiont infections of natural *Chrysoperla carnea* populations. R=*Rickettsia* infection, N= no infection

ID	endosymbiont	Founding place	Date	collector
Chca1	R	Püchau (Wurzen), Saxony	15-07-2015	Gerth et. al.
Chca2	R	Püchau (Wurzen), Saxony	15-07-2015	Gerth et. al.
Chca3	N	Püchau (Wurzen), Saxony	15-07-2015	Gerth et. al.
Chca4	N	Püchau (Wurzen), Saxony	15-07-2015	Gerth et. al.
Chca5	N	Püchau (Wurzen), Saxony	15-07-2015	Gerth et. al.
Chca6	R	Püchau (Wurzen), Saxony	15-07-2015	Gerth et. al.
Chca7	R	Püchau (Wurzen), Saxony	15-07-2015	Gerth et. al.
Chca8	R	Püchau (Wurzen), Saxony	15-07-2015	Gerth et. al.
Chca9	N	Püchau (Wurzen), Saxony	15-07-2015	Gerth et. al.
Chca10	R	Püchau (Wurzen), Saxony	15-07-2015	Gerth et. al.
Chca11	R	Püchau (Wurzen), Saxony	15-07-2015	Gerth et. al.
Chca12	R	Püchau (Wurzen), Saxony	15-07-2015	Gerth et. al.
Chca13	R	Püchau (Wurzen), Saxony	15-07-2015	Gerth et. al.
Chca14	R	Püchau (Wurzen), Saxony	15-07-2015	Gerth et. al.
Chca15	N	Püchau (Wurzen), Saxony	15-07-2015	Gerth et. al.
Chca16	R	Püchau (Wurzen), Saxony	15-07-2015	Gerth et. al.
Chca17	R	Püchau (Wurzen), Saxony	15-07-2015	Gerth et. al.
Chca18	R	Püchau (Wurzen), Saxony	15-07-2015	Gerth et. al.
Chca19	R	Püchau (Wurzen), Saxony	15-07-2015	Gerth et. al.
CCar1	R	Trages, Saxony	17-06-2012	Gerth
CCar2	N	Trages, Saxony	2010-2011	Wolf et al.
CCar3	N	Trages, Saxony	2010-2011	Wolf et al.
CCar4	N	Trages, Saxony	2010-2011	Wolf et al.
CCar5	R	Trages, Saxony	2010-2011	Wolf et al.
CCar6	R	Dahlen, Saxony	22-28-07-2012	Bleidorn et al.
CCar7	N	Dahlen, Saxony	22-28-07-2012	Bleidorn et al.
CCar8	N	Dahlen, Saxony	22-28-07-2012	Bleidorn et al.
CCar9	N	Dahlen, Saxony	22-28-07-2012	Bleidorn et al.
CCar10	R	Dahlen, Saxony	22-28-07-2012	Bleidorn et al.
CCar12	R	Dahlen, Saxony	22-28-07-2012	Bleidorn et al.
CCar14	R	Dahlen, Saxony	22-28-07-2012	Bleidorn et al.
CCar15	R	Dahlen, Saxony	22-28-07-2012	Bleidorn et al.
CCar17	R	Dahlen, Saxony	22-28-07-2012	Bleidorn et al.
CCar18	R	Dahlen, Saxony	22-28-07-2012	Bleidorn et al.
CCar19	N	Dahlen, Saxony	22-28-07-2012	Bleidorn et al.
CCar20	N	Dahlen, Saxony	22-28-07-2012	Bleidorn et al.

CCar21	N	Dahlen, Saxony	22-28-07-2012	Bleidorn et al.
CCar22	N	Dahlen, Saxony	22-28-07-2012	Bleidorn et al.
CCar24	R	Neudorf, Saxony	21-27-07-2012	Gerth et. al.
CCar25	N	Neudorf, Saxony	21-27-07-2012	Gerth et. al.
CCar26	R	Neudorf, Saxony	21-27-07-2012	Gerth et. al.
CCar27	N	Neudorf, Saxony	21-27-07-2012	Gerth et. al.
CCar28	N	Neudorf, Saxony	21-27-07-2012	Gerth et. al.
CCar29	N	Neudorf, Saxony	21-27-07-2012	Gerth et. al.
BK1	R	Kranzberg, Bavaria	24-08-2015	Gruppe
BK2	R	Kranzberg, Bavaria	24-08-2015	Gruppe
BK3	R	Kranzberg, Bavaria	24-08-2015	Gruppe
BK4	N	Kranzberg, Bavaria	24-08-2015	Gruppe
BK5	R	Kranzberg, Bavaria	24-08-2015	Gruppe
BK6	R	Kranzberg, Bavaria	24-08-2015	Gruppe
BK7	R	Kranzberg, Bavaria	24-08-2015	Gruppe
BK8	R	Kranzberg, Bavaria	24-08-2015	Gruppe
BK9	R	Kranzberg, Bavaria	24-08-2015	Gruppe
BK10	R	Kranzberg, Bavaria	24-08-2015	Gruppe
BK11	R	Kranzberg, Bavaria	24-08-2015	Gruppe
BK12	R	Kranzberg, Bavaria	24-08-2015	Gruppe
BS1	R	Schönau, Bavaria	27-08-2015	Gruppe
BS2	R	Schönau, Bavaria	27-08-2015	Gruppe
BS3	N	Schönau, Bavaria	27-08-2015	Gruppe
BS4	N	Schönau, Bavaria	27-08-2015	Gruppe
BS5	R	Schönau, Bavaria	27-08-2015	Gruppe
BS6	N	Schönau, Bavaria	27-08-2015	Gruppe
BS7	N	Schönau, Bavaria	27-08-2015	Gruppe
BS8	R	Schönau, Bavaria	27-08-2015	Gruppe
BW1	R	Wippenhausen, Bavaria	12-08-2015	Gruppe
BW2	R	Wippenhausen, Bavaria	12-08-2015	Gruppe
BW3	N	Wippenhausen, Bavaria	12-08-2015	Gruppe
BW4	R	Wippenhausen, Bavaria	12-08-2015	Gruppe
BW5	N	Wippenhausen, Bavaria	12-08-2015	Gruppe
BW6	R	Wippenhausen, Bavaria	12-08-2015	Gruppe
BW7	N	Wippenhausen, Bavaria	12-08-2015	Gruppe
BW8	N	Wippenhausen, Bavaria	12-08-2015	Gruppe
BW9	R	Wippenhausen, Bavaria	12-08-2015	Gruppe
BW10	R	Wippenhausen, Bavaria	12-08-2015	Gruppe
BW11	N	Wippenhausen, Bavaria	12-08-2015	Gruppe
BW12	R	Wippenhausen, Bavaria	12-08-2015	Gruppe
AV1	R	Rankweil, Vorarlberg, Austria	5-7-8-2015	Gruppe

AV2	R	Rankweil, Vorarlberg, Austria	5-7-8-2015	Gruppe
AV3	R	Rankweil, Vorarlberg, Austria	5-7-8-2015	Gruppe
AV4	N	Rankweil, Vorarlberg, Austria	5-7-8-2015	Gruppe
AV5	R	Rankweil, Vorarlberg, Austria	5-7-8-2015	Gruppe
AV6	R	Rankweil, Vorarlberg, Austria	5-7-8-2015	Gruppe
AV7	R	Rankweil, Vorarlberg, Austria	5-7-8-2015	Gruppe
AV8	R	Rankweil, Vorarlberg, Austria	5-7-8-2015	Gruppe

Table S2 used primers of PCR and qPCR.

Gene region	Primer name	Direction	Primer (5' → 3')	Amplicon size	Reference
Rickettsia 16S-rRNA	Rb_F	Forward	GCTCAGAACGAACGCTATC	963bp	[52]
	Rb_R	Reverse	GAAGGAAAGCATCTCTGC		[52]
Sodalis 16S-rRNA	Sodalis_CC_16S_F	Forward	CGGTGCCGTAAATAGCGC	1059bp	This paper
	Sodalis_16SB1	Reverse	TACGGYTACCTTGTTACGACTT		[80]
Actin	ActinQ1	Forward	TCCAGAAGAACACCCAATCC	188bp	[51] 52
	ActinQ2	Reverse	ATACACCATCACCAGAGTCAAGT		[51] 52
Rickettsia-gltA	gltA-R Fw	Forward	TCGGCACATCAAGAGTTAAA	222bp	This paper
	gltA-R Rv	Reverse	TTGCGTTAGTTTTCATCGTG		This paper
Sodalis-groEL	groEL-S F	Forward	CAGTTCGATCGTGGTTATCT	182bp	This paper
	groEL-S R	Reverse	CTTCAGCAATAATCAGCAGC		This paper

Table S3 Accession numbers of sequences used for the phylogenetic placement of the green lacewing *Sodalis* strain

Table S3 Accession numbers of sequences used for the phylogenetic placement of the green lacewing *Sodalis* strain.

Accession number	Name of sequence
**outgroups**	
**AB626123**	*Proteus mirabilis* gene for 16S rRNA, partial sequence
**AY660027**	*Xenorhabdus bovienii* 16S ribosomal RNA gene
**EF467859**	*Photorhabdus temperata* subsp. *stackebrandtii* strain GPS11 16S ribosomal RNA gene
**HM196339**	*Brenneria quercina* strain W3L22 16S ribosomal RNA gene
**HM585373**	*Serratia rubidaea* strain E9 16S ribosomal RNA gene
**J01874.**	*Proteus vulgaris* 16S ribosomal RNA
Sodalis	
**AB507712**	Secondary endosymbiont of *Curculio sikkimensis* gene for 16S ribosomal RNA
**AB604872**	*Sodalis* secondary endosymbiont of *Archarius roelofsi* gene for 16S ribosomal RNA

**AB604873**	*Sodalis* secondary endosymbiont of *Curculio hachijoensis* gene for 16S ribosomal RNA
**AB772196**	*Sodalis*-like endosymbiont of *Philaenus spumarius* gene for 16S ribosomal RNA
**AB809072**	*Sodalis*-like secondary symbiont of *Antestiopsis thunbergii* gene for 16S ribosomal RNA
**AB915770**	*Sodalis* endosymbiont of *Elasmucha putoni* gene for 16S ribosomal RNA
**AB915771**	*Sodalis* endosymbiont of *Aelia fieberi* gene for 16S ribosomal RNA
**AB915773**	*Sodalis* endosymbiont of *Dolycoris baccarum* gene for 16S ribosomal RNA
**AB915774**	*Sodalis* endosymbiont of *Glaucias subpunctatus* gene for 16S ribosomal RNA
**AB915775**	*Sodalis* endosymbiont of *Lelia decempunctata* gene for 16S ribosomal RNA
**AB915776**	*Sodalis* endosymbiont of *Nezara antennata* gene for 16S ribosomal RNA
**AB915779**	*Sodalis* endosymbiont of *Palomena angulosa* gene for 16S ribosomal RNA
**AB915780**	*Sodalis* endosymbiont of *Picromerus lewisi* gene for 16S ribosomal RNA
**AB915781**	*Sodalis* endosymbiont of *Piezodorus hybneri* gene for 16S ribosomal RNA
**AB915782**	*Sodalis* endosymbiont of *Poecilocoris lewisi* gene for 16S ribosomal RNA
**AB915783**	*Sodalis* endosymbiont of *Rhopalus sapporensis* gene for 16S ribosomal RNA
**AF005235**	*Sitophilus oryzae* principal endosymbiont 16S ribosomal RNA gene
**AM774412**	*Biostraticola tofi* partial 16S rRNA gene, type strain BF36T
**AM946408**	Uncultured bacterium partial 16S rRNA gene, isolated from *Tetropium castaneum*
**AY126638**	Primary endosymbiont of *Sitophilus granarius* 16S ribosomal RNA gene
**CP006568**	Candidatus Sodalis pierantonius
**DQ115535**	Primary endosymbiont of *Pseudolynchia canariensis* 16S ribosomal RNA gene
**EF174495**	*Sodalis* endosymbiont of *Craterina melbae* strain CMS06 16S ribosomal RNA gene
**JN872637**	Candidatus Sodalis melophagi strain CZT 16S ribosomal RNA gene
**JN872639**	Bacterium endosymbiont of *Ornithomya avicularia* 16S ribosomal RNA gene
**JQ063439**	Endosymbiont of *Columbicola columbae* strain 07.15.02 16S ribosomal RNA gene
**JX444565**	*Sodalis praecaptivus* strain HS
**KX146203**	Uncultured bacterium clone Axsp714095.2 16S ribosomal RNA gene
**M85269**	Endosymbiont *Sitophilus zeamais* 16S ribosomal RNA
**M99060**	*Glossina pallidipes* S-endosymbiont (tsetse) endosymbiont ribosomal RNA small subunit
**MF429885**	*Sodalis* endosymbiont of *Icosta* sp. 2 ES-2018 clone SeP5 16S ribosomal RNA gene
**MF429888**	*Sodalis* endosymbiont of *Icosta* sp. 4 ES-2018 clone SeWAN 16S ribosomal RNA gene
**NR074525**	*Sodalis glossinidius* str. '*morsitans*' strain *morsitans* 16S ribosomal RNA
**U64867**	*Glossina palpalis* S-endosymbiont 16S ribosomal RNA gene
**U64868**	*Glossina fuscipes* S-endosymbiont 16S ribosomal RNA gene
**U64869**	*Glossina austeni* S-endosymbiont 16S ribosomal RNA gene
**U64870**	*Glossina brevipalpis* S-endosymbiont 16S ribosomal RNA gene

Table S4 Number of laid eggs, viable larvae, pupae and emerged adults of uninfected (N), only *Rickettsia* (R), only *Sodalis* (S) or combined infected (RS) *Chrysperla carnea*

Endosymbiont	eggs	larvae	pupae	adults	Endosymbiont	eggs	larvae	pupae	adults
R	114	51	15	10	S	70	20	5	4
R	268	123	24	21	S	103	13	9	5
R	116	17	4	4	S	49	6	0	0
R	141	59	20	13	S	136	60	20	19
R	152	72	21	17	S	117	28	7	7
R	136	55	12	11	S	79	47	18	14

R	227	90	21	19	S	37	1	0	0
RS	32	10	2	2	S	62	23	5	5
RS	13	1	0	0	N	158	84	28	27
RS	13	3	2	1	N	136	83	29	25
RS	21	0	0	0	N	156	87	26	24
RS	149	69	7	5	N	130	45	15	13
RS	151	32	1	1	N	124	68	30	27
RS	50	10	7	5					
RS	79	24	5	5					
RS	63	30	17	12					
RS	48	61	15	13					

Table S5 Relative quantity of *Rickettsia* and *Sodalis* endosymbionts in *Chrysoperla carnea* using a specific *Rickettsia* (*gltA*) and *Sodalis* (*groEL*) gene fragment in only *Rickettsia* (R), only *Sodalis* (S) or co-infected (RS) adults (a) or larvae (l) revealed by qPCR.

endosymbiont	stage	code	*gltA*	*groEL*
Rickettsia	Adult	aR	0.08	n.d.
Rickettsia	Adult	aR	0.12	n.d.
Rickettsia	Adult	aR	0.57	n.d.
Rickettsia	Adult	aR	0.21	n.d.
Rickettsia	Adult	aR	0.23	n.d.
Rickettsia	Adult	aR	0.15	n.d.
Rickettsia	Adult	aR	0.06	n.d.
Rickettsia	Adult	aR	0.11	n.d.
Rickettsia	Adult	aR	0.11	n.d.
Sodalis	Adult	aS	n.d.	155.96
Sodalis	Adult	aS	n.d.	98.36
Sodalis	Adult	aS	n.d.	57.88
Sodalis	Adult	aS	n.d.	65.80
Sodalis	Adult	aS	n.d.	30.38
Sodalis	Adult	aS	n.d.	60.20
Sodalis	Adult	aS	n.d.	80.17
Sodalis	Adult	aS	n.d.	47.18
Sodalis	Adult	aS	n.d.	86.52
Rickettisa + *Sodalis*	Adult	aRS	0.15	288.02
Rickettisa + *Sodalis*	Adult	aRS	0.25	100.78
Rickettisa + *Sodalis*	Adult	aRS	0.09	237.21
Rickettisa + *Sodalis*	Adult	aRS	0.06	149.09
Rickettisa + *Sodalis*	Adult	aRS	0.18	82.14
Rickettisa + *Sodalis*	Adult	aRS	0.13	172.45
Rickettisa + *Sodalis*	Adult	aRS	0.23	88.04
Rickettisa + *Sodalis*	Adult	aRS	0.22	85.04

Rickettisa + *Sodalis*	Larvae	lRS	4.23	0.03
Rickettisa + *Sodalis*	Larvae	lRS	4.20	0.04
Rickettisa + *Sodalis*	Larvae	lRS	8.72	0.07
Rickettisa + *Sodalis*	Larvae	lRS	3.80	0.03
Rickettisa + *Sodalis*	Larvae	lRS	7.16	0.10
Rickettisa + *Sodalis*	Larvae	lRS	3.54	0.07
Rickettisa + *Sodalis*	Larvae	lRS	3.88	0.08
Rickettisa + *Sodalis*	Larvae	lRS	2.98	0.10
Rickettisa + *Sodalis*	Larvae	lRS	2.36	0.01
Rickettisa + *Sodalis*	Larvae	lRS	1.72	0.02
